# Supplementary material for: Calibration of transmission-dynamic infectious disease models: A scoping review and reporting framework
Source: PLoS Comput Biol. 2025 Nov 4;21(11):e1013647. doi: 10.1371/journal.pcbi.1013647 (PMC12604776; doi:10.1371/journal.pcbi.1013647)
Supplement: S1 Table — (DOCX) [file pcbi.1013647.s001.docx]

**S1 Table*:*** *Inclusion and exclusion criteria based on the Studies, Data, Methods and Outcome (SDMO) framework.*

| **SDMO** | **Element** | **Inclusion and exclusion criteria** |
| --- | --- | --- |
| **Studies** | Dynamic transmission models of HIV, TB or malaria in humans | Include:   - Published dynamic transmission modeling studies of HIV, TB or malaria in humans published from 1 January 2018 to 16 January 2024 (search date).   Exclude:   - Studies based on models that do not have a dynamic transmission component. - Studies in which no model is proposed. - Studies focused on diseases other than HIV, TB or malaria. - Studies that do not involve human populations. - Studies not available in the English language. |
| **Data** | Empirical data or published estimates. | Include:   - Studies which calibrate models to empirical data or published estimates.   ­­­­­­­­Exclude:   - Studies which calibrate models to simulated data. - Studies for which the data used is not mentioned or described. |
| **Method** | Model calibration | Include:   - Studies which calibrate their models to empirical data or published estimates.   Exclude   - Studies that do not perform model calibration. |
| **Outcomes** |  | Outcome measures in studies do not form part of the inclusion or exclusion criteria. |
