# Supplementary material for: Calibration of transmission-dynamic infectious disease models: A scoping review and reporting framework
Source: PLoS Comput Biol. 2025 Nov 4;21(11):e1013647. doi: 10.1371/journal.pcbi.1013647 (PMC12604776; doi:10.1371/journal.pcbi.1013647)
Supplement: S3 Table — (DOCX) [file pcbi.1013647.s003.docx]

**S3 Table**: *Full electronic search* strategy*

| Database (Platform) | Search | Results |
| --- | --- | --- |
| pubmed (nlm) | (("Epidemiological Models"[Mesh] OR ("Transmission model"[tiab:~3] OR "transmission models"[tiab:~3] OR "dynamic model"[tiab:~3] OR "dynamic models"[tiab:~3] OR "mathematical model"[tiab:~3] OR "mathematical models"[tiab:~3] OR "simulation model"[tiab:~3] OR "Simulation models"[tiab:~3])) AND ("Malaria"[Mesh] OR malaria*[tiab] OR "plasmodium infection*"[tiab] OR "remittent fever*"[tiab] OR "Marsh fever*"[tiab] OR paludis*[tiab] OR "Tuberculosis"[Mesh] OR tuberculos*[tiab] OR tuberculoma*[tiab] OR koch-disease*[tiab] OR "HIV infections"[Mesh] OR "HIV"[Majr] OR HIV[tiab] OR "human immunodeficiency virus"[tiab] OR "Human immuno-deficiency virus"[tiab] OR HIVAIDS[tiab] OR "acquired immunodeficiency syndrome*"[tiab] OR "acquired immuno-deficiency syndrome*"[tiab])) NOT (“animals”[mesh] NOT “Humans”[mesh]) **Date Range: from 1 January 2018 – 31 December 2023** | 1215 |
| embase (elsevier) | 1. ((‘Disease model’/exp/mj OR ‘Mathematical model’/exp/mj) AND ('disease transmission'/exp OR 'infection control'/exp)) OR ((transmission* OR dynamic* OR mathematical* OR simulation*) NEAR/3 model*):ti,ab,kw    2. 'Malaria'/exp OR 'Human immunodeficiency Virus'/exp/mj OR 'Human immunodeficiency virus infections'/exp OR 'Tuberculosis'/exp OR ('human immunodeficiency virus' OR HIV OR 'human immuno-deficiency virus' OR HIVAIDS OR 'acquired immunodeficiency sydrome*' OR 'acquired immuno-deficiency syndrome*' OR malaria* OR tuberculos* OR remittent-fever* OR marsh-fever* OR paludis* OR tuberculoma OR koch-disease*):ti,ab,kw  3. (('Nonhuman'/syn OR 'Animal'/syn) NOT 'Human'/exp)    4. #1 AND #2 NOT #3 AND [embase]/lim AND [2018-2023]/py AND ([article]/lim OR [article in press]/lim OR [data papers]/lim OR [review]/lim OR [preprint]/lim) | 1236 |
| Global Health (ebsco) | 1. DE (("disease models” OR "mathematical models" OR "simulation models") AND ("transmission" OR "infection control" OR "infectious diseases")) OR TI ((dynamic* OR transmission* OR mathematical* OR simulation*) N3 model*) OR AB ((dynamic* OR transmission* OR mathematical* OR simulation*) N3 model*)  2. DE ("malaria" OR "Blackwater fever" OR "Tuberculosis" OR "extrapulmonary tuberculosis" OR "miliary tuberculosis" OR "Human immunodeficiency viruses" OR "HIV Infections") OR TI ("human immunodeficiency virus" OR HIV OR "human immuno-deficiency virus" OR HIVAIDS OR "acquired immunodeficiency sydrome*" OR "acquired immuno-deficiency syndrome*" OR malaria* OR tuberculos* OR remittent-fever* OR marsh-fever* OR paludis* OR tuberculoma OR koch-disease*) OR AB ("human immunodeficiency virus" OR HIV OR "human immuno-deficiency virus" OR HIVAIDS OR "acquired immunodeficiency sydrome*" OR "acquired immuno-deficiency syndrome*" OR malaria* OR tuberculos* OR remittent-fever* OR marsh-fever* OR paludis* OR tuberculoma OR koch-disease*)  S1 AND S2  Limiters - Publication Year: 20180101-20231231 | 1367 |
| Web of science core collection – SCI-EXP & ESCI (Clarivate) | #1 (TI=(model* NEAR/3 (mathematical* OR simulation$ OR transmission OR dynamic$))) OR (AB=(model* NEAR/3 (mathematical* OR simulation$ OR transmission OR dynamic$))) OR (AK=(model* NEAR/3 (mathematical OR simulation$ OR transmission OR dynamic$)))  #2 (TI=("human immunodeficiency virus" OR HIV OR "human immuno-deficiency virus" OR HIVAIDS OR "acquired immunodeficiency sydrome*" OR "acquired immuno-deficiency syndrome*" OR malaria* OR tuberculos* OR remittent-fever* OR marsh-fever* OR paludis* OR tuberculoma OR koch-disease*)) OR (AB=("human immunodeficiency virus" OR HIV OR "human immuno-deficiency virus" OR HIVAIDS OR "acquired immunodeficiency sydrome*" OR "acquired immuno-deficiency syndrome*" OR malaria* OR tuberculos* OR remittent-fever* OR marsh-fever* OR paludis* OR tuberculoma OR koch-disease*)) OR (AK=("human immunodeficiency virus" OR HIV OR "human immuno-deficiency virus" OR HIVAIDS OR "acquired immunodeficiency sydrome*" OR "acquired immuno-deficiency syndrome*" OR malaria* OR tuberculos* OR remittent-fever* OR marsh-fever* OR paludis* OR tuberculoma OR koch-disease*))    #1 AND #2 AND 2018-2023 | 2359 |
| GLobal Index medicus – all indices (WHO) | tw:((transmission OR dynamic OR simulation OR mathematical) AND model*) AND tw:("human immunodeficiency virus" OR HIV OR "human immuno-deficiency virus" OR HIVAIDS OR "acquired immunodeficiency sydrome*" OR "acquired immuno-deficiency syndrome*" OR malaria* OR tuberculos* OR remittent-fever* OR marsh-fever* OR paludis* OR tuberculoma OR koch-disease*)  Date limit: 2018-2023 | 55 |
| From Websites | | 286 |
| TOTAL | | 6518 |
| After Deduplication | | 3138 |

Grey Literature Search

| Website URL | # of Articles Retrieved |
| --- | --- |
| http://hivmodeling.org/publications-reports | 21 |
| TB-MAC NEWSLETTERS | 265 |
| TOTAL | 286 |

*Searches run on 16 January 2024 by Emmanuelle A Dankwa
