# Supplementary material for: Calibration of transmission-dynamic infectious disease models: A scoping review and reporting framework
Source: PLoS Comput Biol. 2025 Nov 4;21(11):e1013647. doi: 10.1371/journal.pcbi.1013647 (PMC12604776; doi:10.1371/journal.pcbi.1013647)
Supplement: S4 Table — (DOCX) [file pcbi.1013647.s004.docx]

**S4 Table:** *Details of studies with multiple calibrated models.*

| Study DOI | Full Title | Number Of Calibrated Models | |
| --- | --- | --- | --- |
| [https:// doi.org/10.1371/journal.pone.0242595](https://doi.org/10.1371/journal.pone.0242595) | Challenges in estimating HIV prevalence trends and geographical variation in HIV prevalence using antenatal data: Insights from mathematical modelling | | 2 |
| [https://doi.org/10.1097/ede.0000000000001418](https://doi.org/10.1097/EDE.0000000000001418) | The Health and Economic Benefits of Tests That Predict Future Progression to Tuberculosis Disease | | 3 |
| [http://doi.org/10.1097/qad.0000000000002826](http://doi.org/10.1097/QAD.0000000000002826) | Mathematical modelling of the influence of serosorting on the population-level HIV transmission impact of pre-exposure prophylaxis | | 2 |
| <https://doi.org/10.1164/rccm.201907-1289oc> | Comparative Modeling of Tuberculosis Epidemiology and Policy Outcomes in California. | | 3 |
| <https://doi.org/10.1371/journal.pone.0199453> | The emerging health impact of voluntary medical male circumcision in Zimbabwe: An evaluation using three epidemiological models. | | 3 |
