# Supplementary figures and images for: Calibration of transmission-dynamic infectious disease models: A scoping review and reporting framework
Source: PLoS Comput Biol. 2025 Nov 4;21(11):e1013647. doi: 10.1371/journal.pcbi.1013647 (PMC12604776; doi:10.1371/journal.pcbi.1013647)

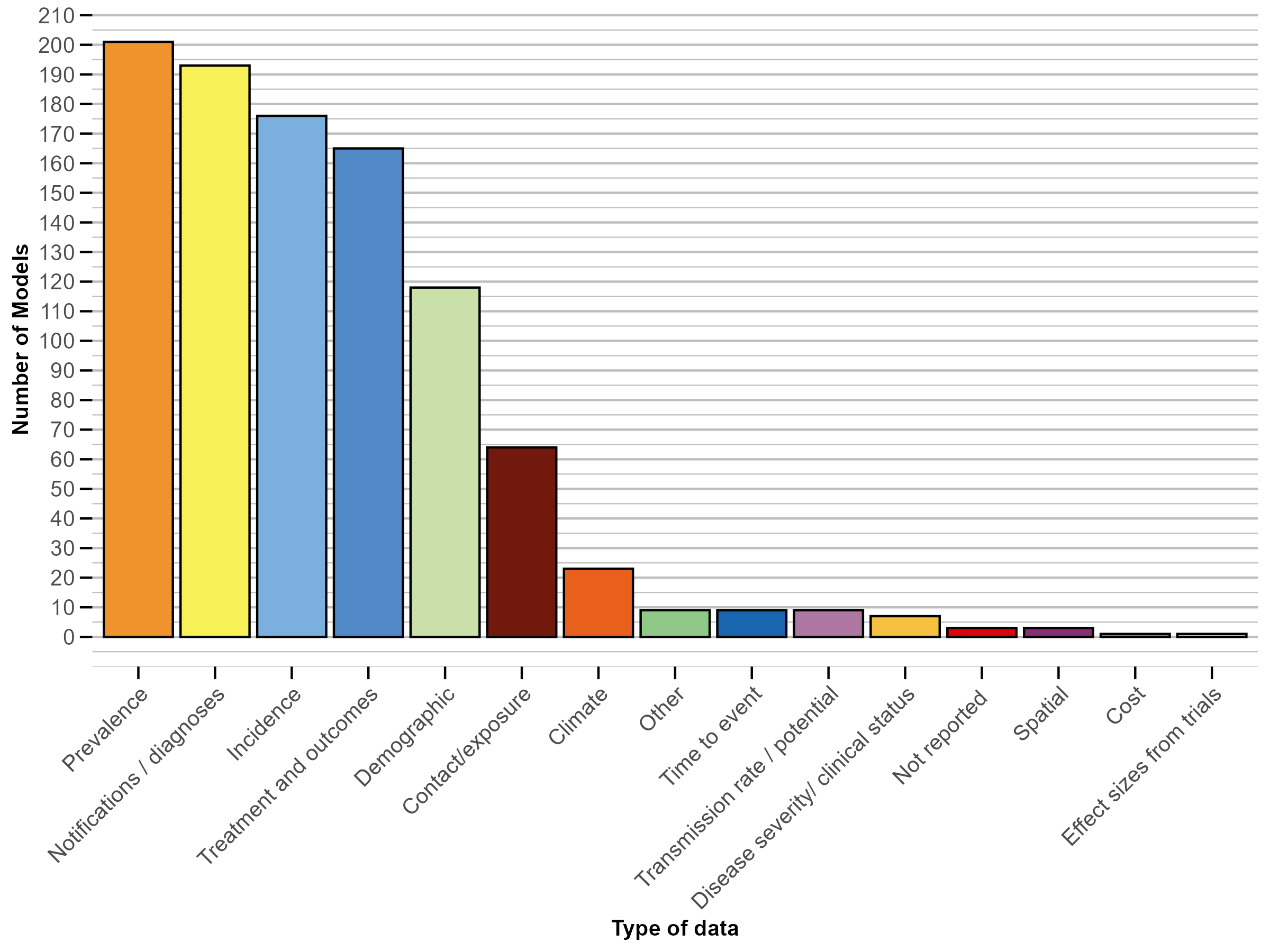

Supplement: S1 Fig — (TIFF) [file pcbi.1013647.s006.tiff]

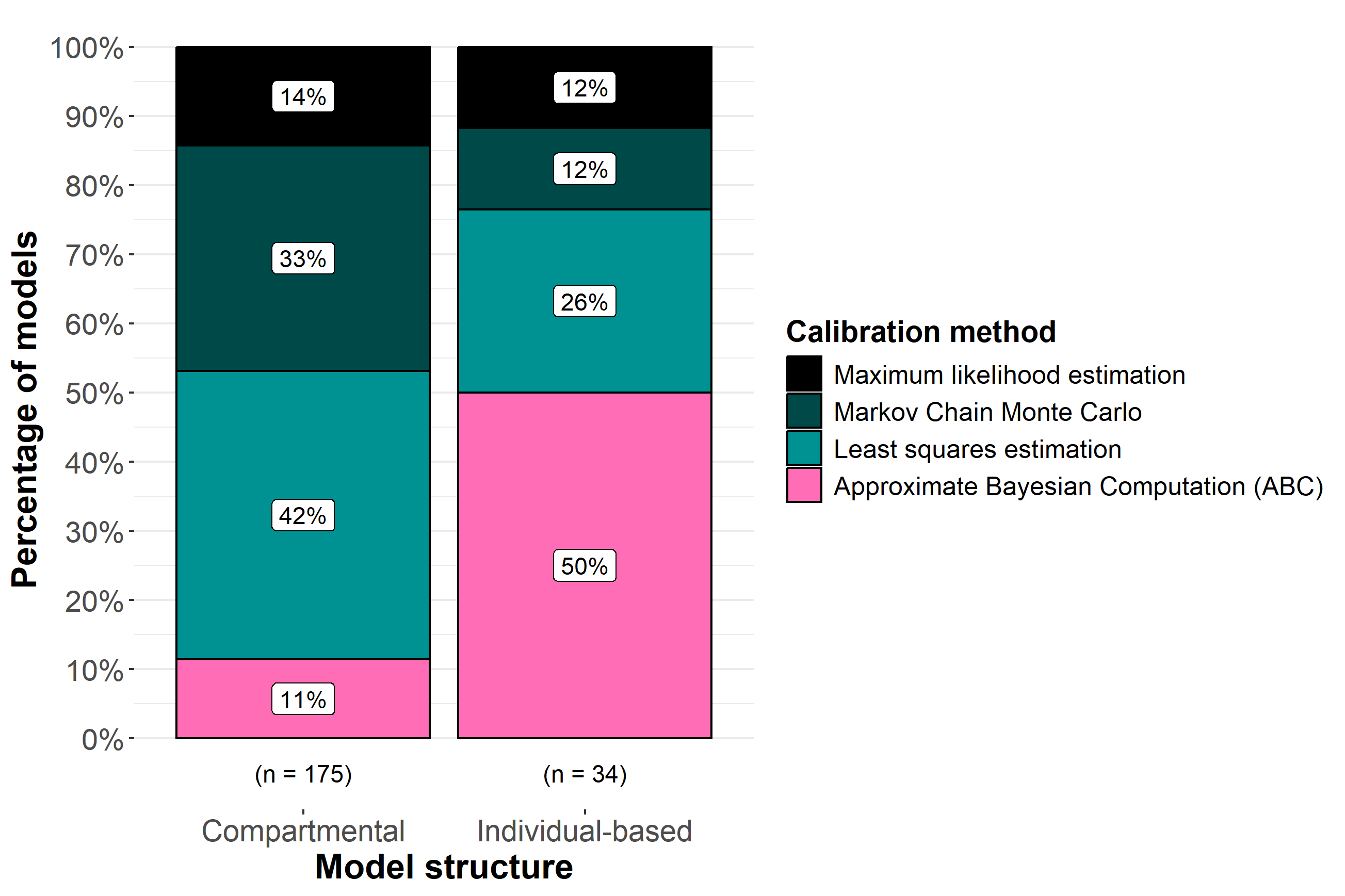

Supplement: S2 Fig — (TIFF) [file pcbi.1013647.s007.tiff]

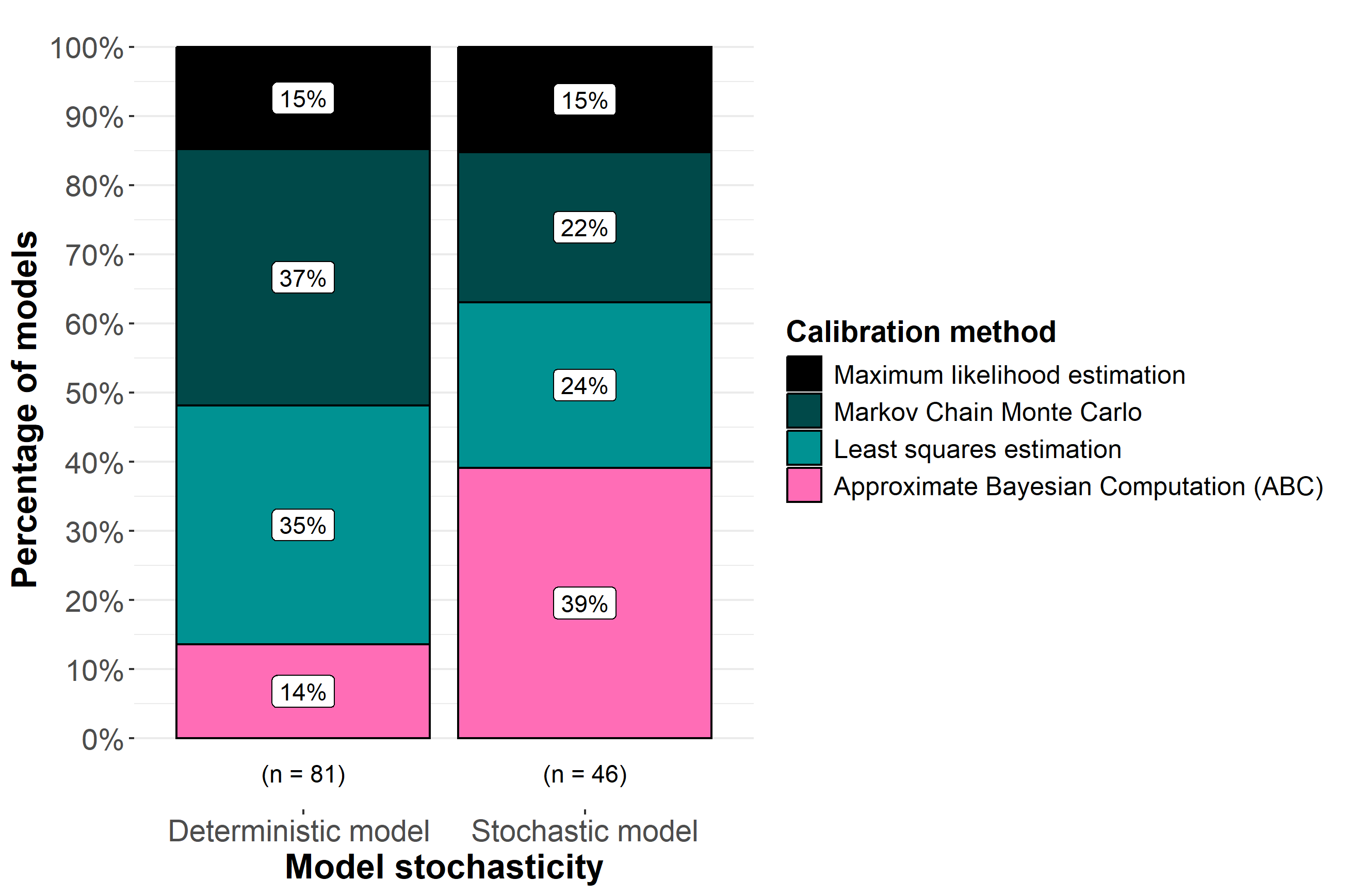

Supplement: S3 Fig — (TIFF) [file pcbi.1013647.s008.tiff]

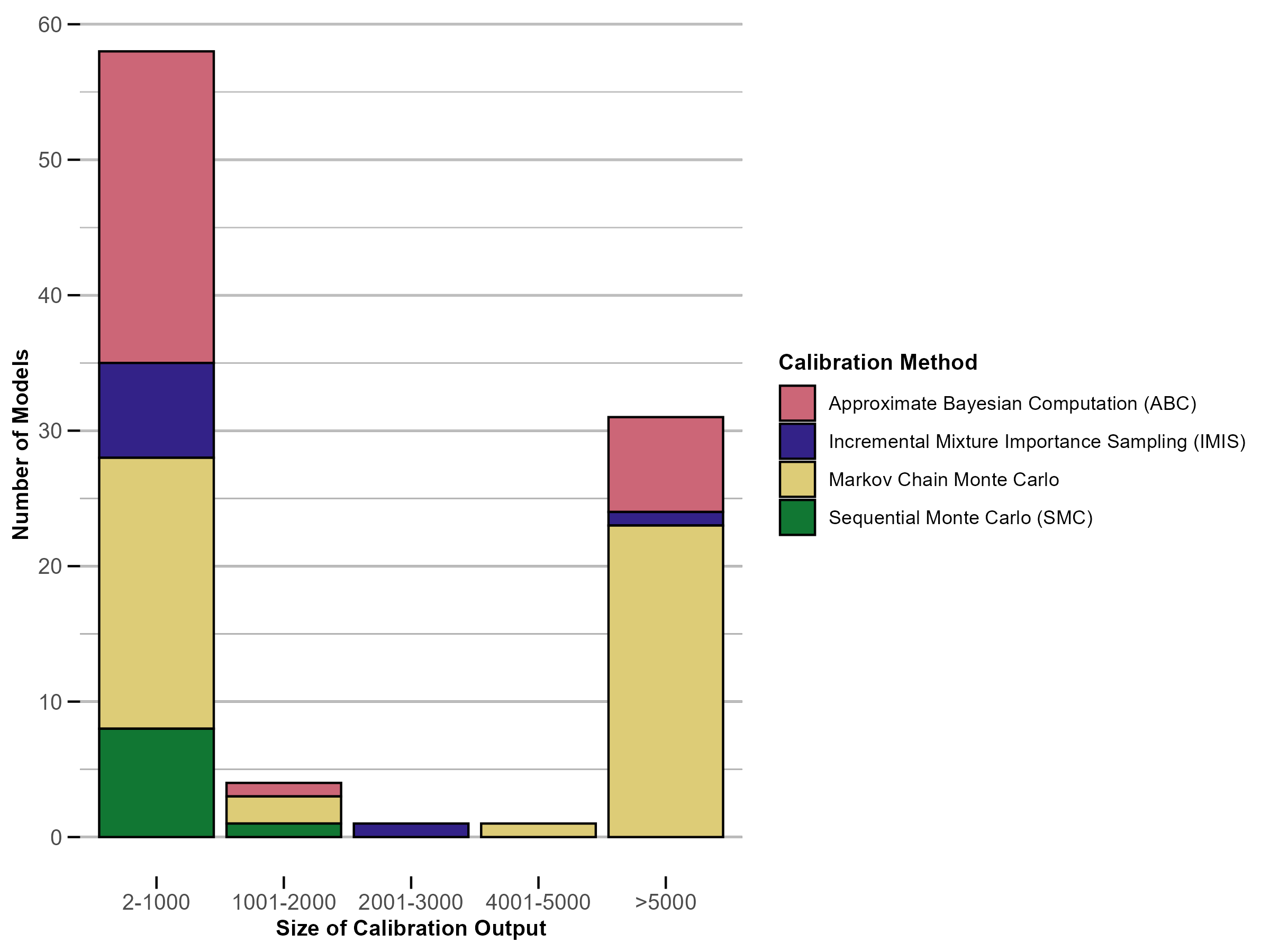

Supplement: S4 Fig — Results are shown for calibration methods reported in at least 10 models. (TIFF) [file pcbi.1013647.s009.tiff]

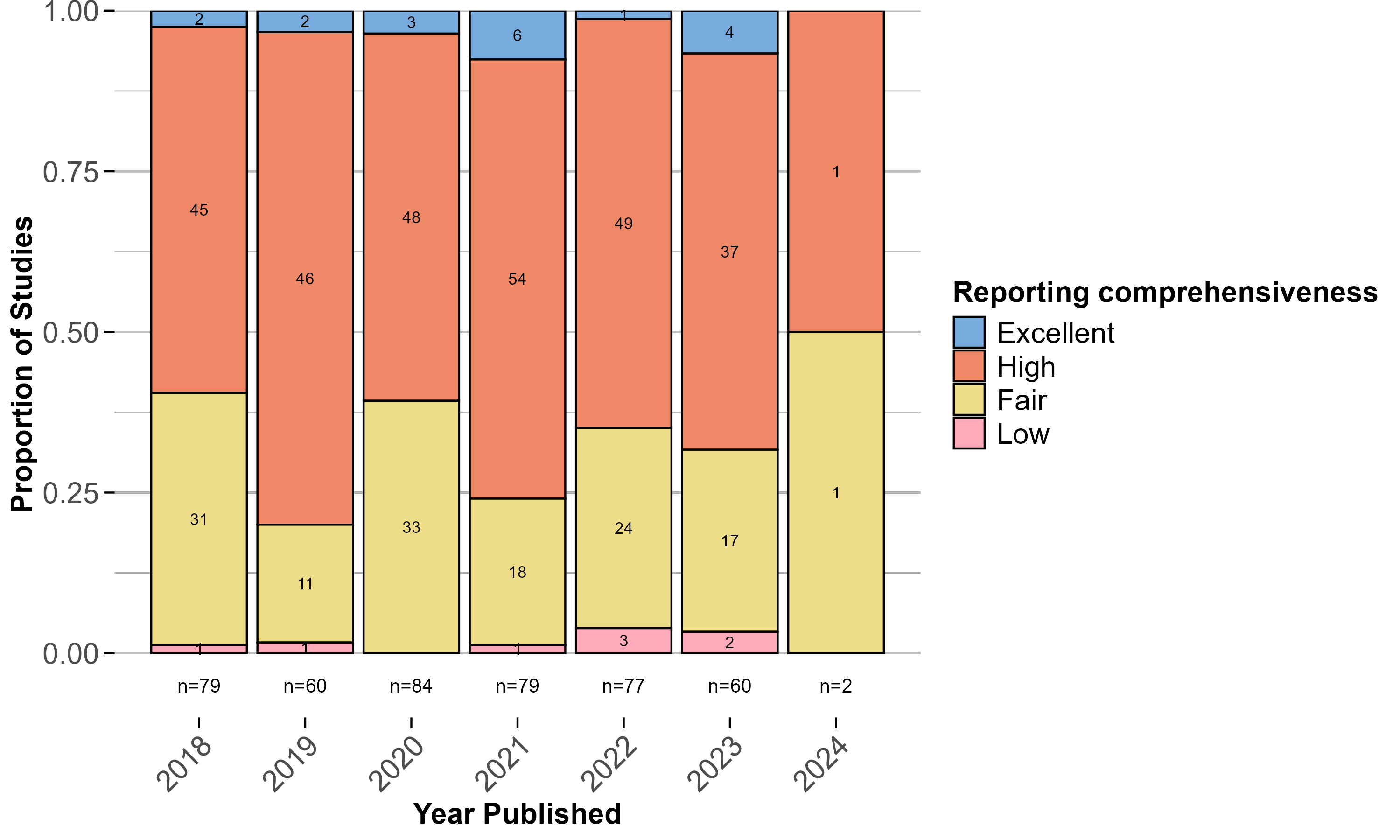

Supplement: S5 Fig — (TIFF) [file pcbi.1013647.s010.tiff]

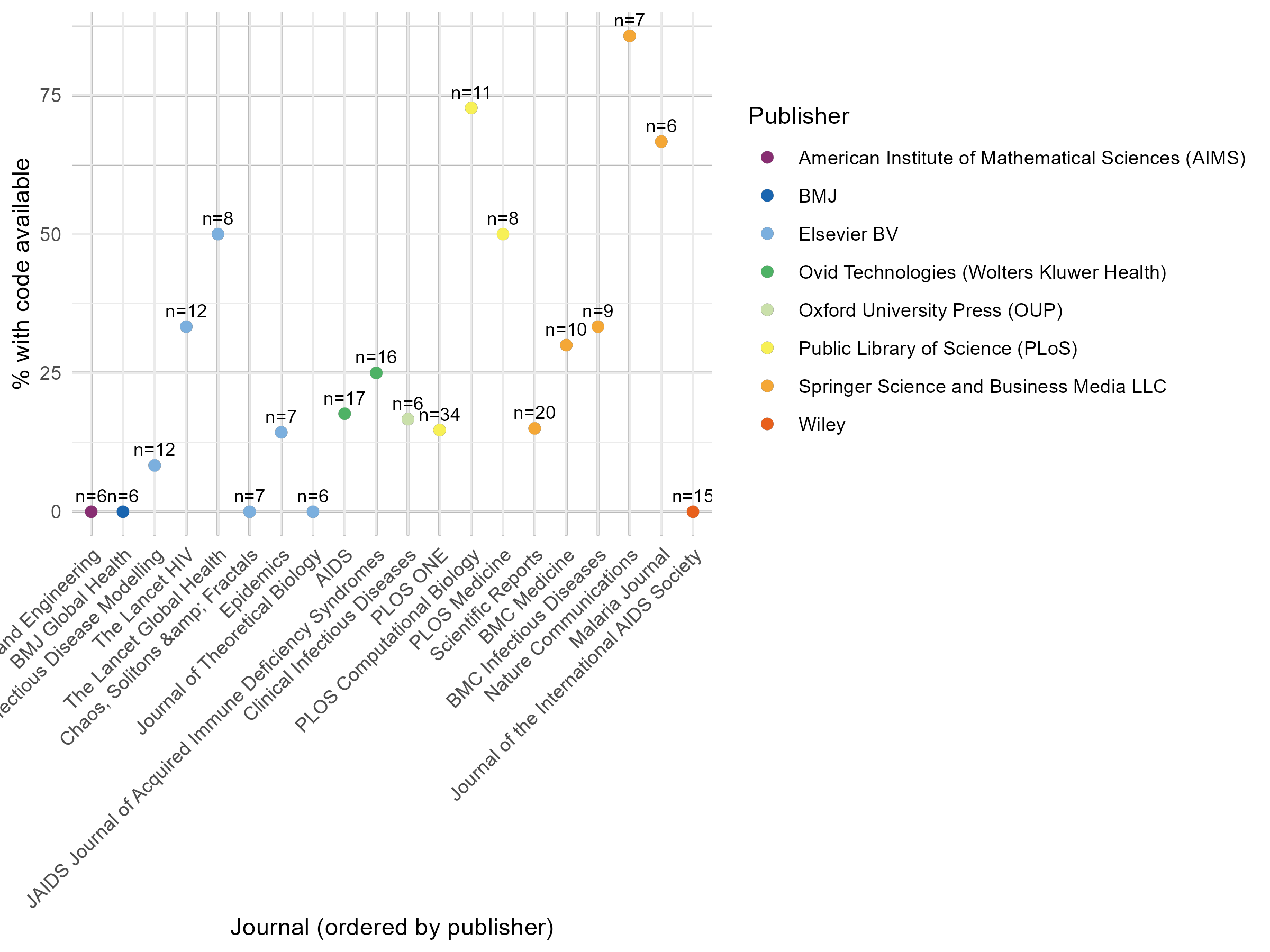

Supplement: S6 Fig — n = number of models per journal in study. Journals are ordered by publisher on the x-axis. Figure is limited to journals with more than five models in the study. (TIFF) [file pcbi.1013647.s011.tiff]

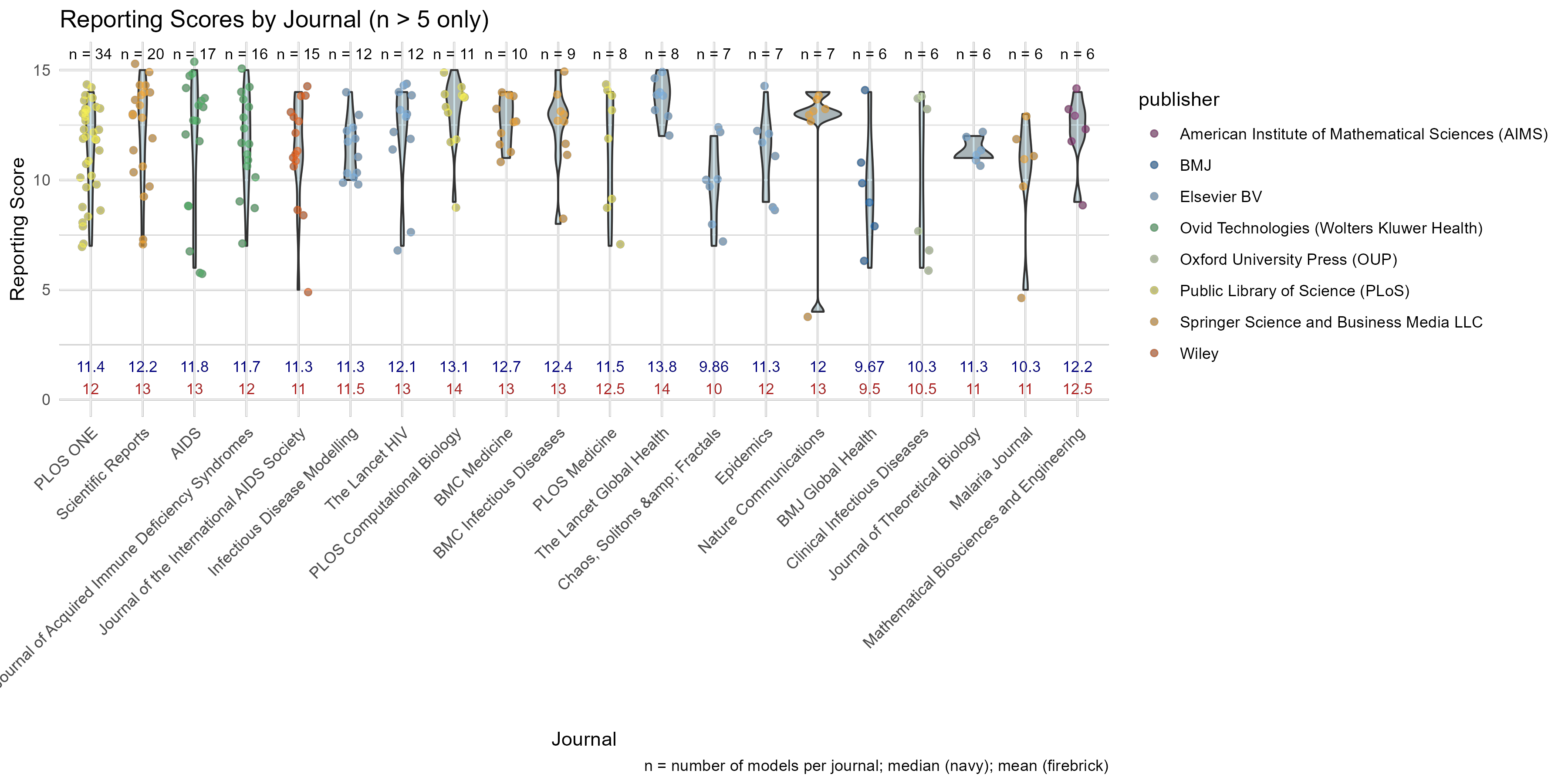

Supplement: S7 Fig — n = number of models per journal in study. Navy numeric annotations indicate the median score per journal while firebrick annotations indicate the mean score. (TIFF) [file pcbi.1013647.s012.tiff]

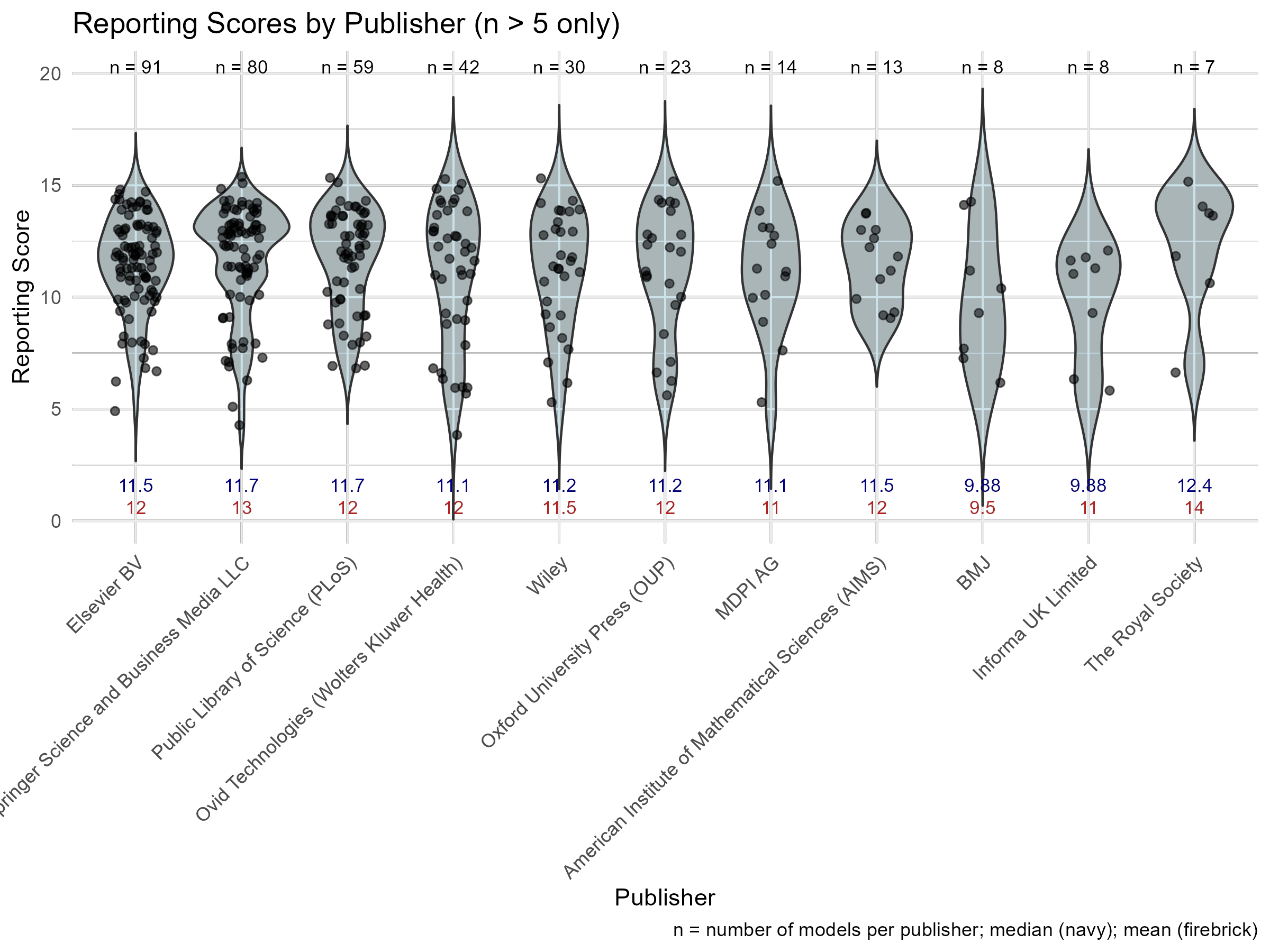

Supplement: S8 Fig — n = number of models per publisher in study. Navy numeric annotations indicate the median score per publisher while firebrick annotations indicate the mean score. (TIFF) [file pcbi.1013647.s013.tiff]
