# Supplementary material for: Calibration of transmission-dynamic infectious disease models: A scoping review and reporting framework
Source: PLoS Comput Biol. 2025 Nov 4;21(11):e1013647. doi: 10.1371/journal.pcbi.1013647 (PMC12604776; doi:10.1371/journal.pcbi.1013647)
Supplement: S1 Text — (DOCX) [file pcbi.1013647.s018.docx]

**PIPO (PURPOSE-INPUTS-PROCESS-OUTPUTS) FRAMEWORK FOR CALIBRATION REPORTING**

Characteristics of calibration methods are classified into four broad components:

1. **Purpose** (*what is the goal of the study?*),
2. **Inputs** (*what are the inputs into the calibration algorithm?*),
3. **Process** (*how is calibration conducted, given inputs?*), and
4. **Outputs** (*what are the characteristics of the calibration outputs?*).

**Table 1: PIPO reporting framework.** Examples describe how calibration methods may differ by item. **Examples are not an exhaustive list**. User should complete last column.

| **ITEM** | **ITEM #** | **EXAMPLES** | **REPORTED ON PAGE NUMBER(S) OR REASON FOR NON-REPORTING** |
| --- | --- | --- | --- |
| **A. *Purpose: what is the goal of the study?*** | | | |
| **A.1. Scientific problem being solved (what is the context of calibration?)** | 1 | Understand disease mechanisms (e.g., through inference on a parameter) |  |
|  |  | Evaluate interventions |  |
|  |  | Predict disease trends |  |
|  |  | Assess impact of model assumptions |  |
| **B. *Inputs: what are the inputs of the calibration algorithm?*** | | | |
| **B.1. Parameter inputs** | | | |
| **B.1.1. Parameters to calibrate.** *Report which parameters are to be calibrated (provide names and descriptions*) *and include the total number of parameters to be calibrated.* | 2 | Names and descriptions for each parameter to be calibrated. |  |
|  |  |  |  |
| **B.1.2. Justification for choice of parameters to calibrate.** | 3 | Parameters are relevant to question of interest. |  |
|  |  | Parameters are uncertain. |  |
| **B.1.3. Prior information used for calibration.** *For each parameter, report on which prior information is used for calibration, if any.* | 4 | A prior estimate or parameter range which may be sourced from previous model estimates (provide references). Examples: mean with a credible/confidence interval or constraints on values for parameter combinations. |  |
|  |  | Expert judgement (provide references) |  |
| **B.2. Calibration targets: the data or estimates to which parameters are calibrated** | | | |
| **B.2.1. Type of data/estimates used for defining calibration targets.** *Provide brief descriptions and references for the data or estimate including any uncertainty estimates associated with calibration targets.* | 5 | Types of data/estimates: incidence, contact/exposure, demography, notifications/diagnoses, prevalence, spatial variables or treatment outcomes.  These data may either be:  a) Empirical (observed) data or statistical summaries of empirical data (e.g., WHO case notification data) or  b) Modeled estimates (e.g., WHO TB incidence estimates). Report on point estimate and any credible or confidence intervals |  |
| **B.2.2. Resolution of data/estimates used for defining calibration targets.** | 6 | Resolution may be in terms of frequency (e.g., weekly or daily data points), spatial coverage (e.g., state- or county-level data) or population stratification (e.g., age-stratified data). |  |
| **B.2.3. Number of calibration targets, number of data points for each calibration target, and any adjustments made to calibration targets.** | 7 | Example: calibration was performed using five calibration targets, each on a weekly time scale over a year.  Adjustments include extrapolation of data and removal of outliers. |  |
| **C. *Process: how is calibration conducted, given inputs?*** | | | |
| **C.1. Number of steps** | 8 | Calibration was done as a single step (i.e., all parameters were calibrated at once). |  |
|  |  | Calibration was done sequentially: different parameters (or subsets of parameters) were calibrated over multiple rounds |  |
| **C.2. Name and description of calibration algorithm.** *State and describe, at least briefly, the calibration algorithm employed.* | 9 | Common algorithms include Markov chain Monte Carlo-based methods, Approximate Bayesian Computation and maximum likelihood estimation. |  |
| **C.3. Calibration implementation.**  *For more guidance on implementation reporting, refer to Section 4 of the Infectious Disease Modeling Reproducibility Checklist (Pokutnaya et al., 2023).* | 10 | Report an accessible repository for code and relevant dependencies (e.g., data, functions, packages, seed, starting values). Ensure that calibration procedure is: 1) implemented in a clearly stated, accessible programming language or software, 2) implementation is well-documented and has meaningful comments, 3) versions of programming language, package or data repository and 4) computational intensity in terms of average runtime-per-simulation or sample. |  |
| **C.4. Goodness-of-fit (GOF) measures employed within calibration algorithm.**  How is *the level of agreement between modeled outcomes and calibration targets measured?* | 11 | Ad-hoc distance function (as in Approximate Bayesian Computation or least squares) |  |
|  |  | Data likelihood |  |
|  |  | Information criterion (e.g., Akaike information criterion, deviance information criterion) |  |
| **D. *Output: what are the characteristics of the calibration outputs?*** | | | |
| **D.1. Nature of calibration output** | 12 | Point estimate (single parameter value/ single parameter set). |  |
|  |  | Samples from a distribution (multiple parameter values/ multiple parameter sets) |  |
|  |  | Parametric distribution (a closed-form distribution function which can generate parameter values, as obtained with variational inference or Laplace approximation). |  |
| **D.2. Reporting of calibration outputs** | 13 | Numerical. Examples:   - numerical parameter estimate - for each calibration target, weight or contribution of the target to the overall goodness of fit, expressed numerically |  |
|  |  | Graphical: Examples:   - a plot of calibrated model trend versus calibration targets - for each calibrated parameter, a 1D marginal distribution plot comparing prior vs posterior or 2D correlation plots comparing parameters |  |
| **D.3. Reporting of uncertainty in calibration output** | 14 | Numerical: As uncertainty intervals around estimates or model outputs. e.g., 95% CI: 1.5 - 4.5 |  |
|  |  | Graphical: As a plot, e.g., a line with shaded areas indicating uncertainty intervals |  |
| **D.4. Size of calibration output**  *Report the number of parameter sets/ parameter values in calibration output.* | 15 | For example, 1000 parameter sets were produced from the calibration. |  |
| **D.5. Model validation:** post-calibration checks to assess model performance for prediction.  *Report on 1) validation targets (data which the model should reproduce, but not used during calibration itself), 2) validation method and 3) validation results.* | 16 |  |  |

From: Dankwa EA et al. “Calibration conduct and reporting in infectious disease dynamic transmission models: a scoping review and reporting framework”.

**References**

Pokutnaya, D. *et al.* (2023) ‘An implementation framework to improve the transparency and reproducibility of computational models of infectious diseases’, *PLOS Computational Biology*, 19(3), p. e1010856. Available at: https://doi.org/10.1371/journal.pcbi.1010856.
